# Some Thoughts on Public Funerals

**Published:** 1912-02-24

**Authors:** 


### SOME THOUGHTS ON PUBLIC FUNERALS.

### A Famous Scientific Surgeon and a Great General Practitioner.

It is well, at a time when politicians, mainly from the absence of sufficient apprehension of the facts and the fulness of knowledge, show a tendency to depart from statesmanship by inflicting needless injustice on the members of the medical profession, that public attention should have been directed to, and, as we believe, fixed upon, the true and better side of medicine as typified in the actual life, work, and death of two surgeons widely differing in the fields of labour in which their energies were expended, but united in the testimony which their funerals afford to the inestimable value to the whole community of the daily service rendered by every faithful member of the profession. On the 16th instant all that was mortal of Lord Lister, who died on February 10, full of years and honours, received at the hands of this and other nations suitable homage and reverence in Westminster Abbey. We are among those who feel that there is still too much of paganism in the outward trappings of funeral ceremonial; but all this in Lord Lister's case was swept aside because of the respect and influence of a great man who was allowed to be consigned to his last resting-place in a plain casket which bore the simple inscription: "Joseph Lister, born April 5, 1827; died February 10, 1912." Surely those who believe that life on earth is a lease for which, at death, God gives us a freehold of greater value may hold the departure from life of a man or a woman to be but an awakening to a higher and better state of existence. So the believer must ever regard many of the garments and trappings of our funeral arrangements as unsuitable, and even revolting. Seeing that the best of the race have ever maintained firmly that "blessed are the dead that die in the Lord," it is clearly wrong, whatever tradition may impose upon us in the way of dress or custom, to take with us to a service like that at Westminster Abbey on the 16th instant, anything short of a thankful and even cheerfull heart. It is no doubt grievous for those who are left behind to be deprived of the example, the counsel, the society, and the encouragement of a great life, rendered precious to them by memories of close association in work or companionship or both. But in the present day, when the lamp of faith in the midst of men and women so often seems so dim, it is well to remember that without faith there can be no inspiration sufficiently strong for the accomplishment of results such as those which have been achieved for the whole of mankind by the devotion, eminent ability, and thoroughness of an exemplar and teacher like Lister.

### Westminster Abbey and our National Life.

That is why Westminster Abbey fulfils such a splendid purpose in the scheme of our national life, for there the last rites of the Church are tendered to the dead with all due solemnity, amidst surroundings which appeal irresistibly to everything which is best in man and woman. The simple inscription on the coffin revealed the modesty of the man, while his greatness and service were commemorated by the service in the Abbey, which was attended spontaneously, as he would have wished, by a large number of his fellow-workers for the sick in the hospitals he loved, from the consulting and active members of the medical staff to the humblest member of the nursing body. They were his pupils and his children. The great world outside was represented by nearly all the ambassadors of foreign countries, voicing the recognition and gratitude of civilised nations; the representatives of education and science; the Prime Minister and politicians of both parties. For the first time, we believe, in history, not only our own King and Royal Family, but the German Emperor were personally represented, and so general was the sense of indebtedness and the wish personally to testify homage to the dead, that it is probable that the wide space within the Abbey walls was hardly sufficient to accommodate all who came with full and grateful hearts to pay their last tribute to the great dead. Like St. Paul's, Westminster Abbey lends itself under its present administration to such an occasion as Lord Lister's funeral. It was not a little remarkable, though perfectly natural and appropriate in the circumstances, and a splendid tribute to the memory of the man and his work, that everyone present was so absorbed with the greatness and solemnity of the occasion that there was a reverent silence throughout and after the service. We welcome as full of hope for far-reaching effects for good this testimony from a gathering which represented in its entirety the best intellect and experience of our day and generation.

### For the Good of the Race.

Last week we bore full testimony to the unanimous sentiment which permeates the hospitals of the entire world in regard to Lord Lister's services. It is a notable tribute to the spirit of medicine, as expressed in the work of members of the medical profession, that the whole world should unite in homage to his greatness and in testimony of its affectionate gratitude for his work. There is nothing remarkable in this; that the work of a man which is computed to have saved more lives than were sacrificed in the whole of the devastating wars of the nineteenth century, should exhibit this great annealing power amongst the living. It would have been remarkable were it otherwise. Lister has worthily been laid to rest. His memory will for ever remain with the workers who give their lives to the endeavour to prolong human life, to mitigate and diminish human suffering, and to make the world a better and happier place for the most miserable of mankind. Lister's work will be a beacon light for centuries, and a living monument for all time. But the service at Westminster Abbey, apart from its historical importance, should drive home to the consciousness of the nation what in God's mercy the greatest of surgeons has been permitted to accomplish for the race.

A Great General Practitioner.

Almost contemporary with Lister, and zealously working during the whole of the period in which Lister was fulfilling his destiny by revolutionising surgical treatment, a great general practitioner of the old school, the descendant of a family of surgeons who for generations had rendered inestimable service to the inhabitants of one district of Wiltshire, was in his way revolutionising medical treatment and giving a new hope and prolonged life to hundreds of the poorer people in Marlborough and a wide district around it. On Saturday, February 17, the day after the funeral of Lord Lister in Westminster Abbey, Dr. James Blake Maurice was buried in Preshute Churchyard, Wilts. Dr. Maurice took the fellowship of the College of Surgeons by examination in 1864, some ten years later than Lister. After refusing the opening offered him to join the staff of a great London hospital, and making a thorough study of German and other foreign clinics, he joined his father in Marlborough and began his career as a general practitioner. A few years earlier, in 1859, Albert Napper had opened the first cottage hospital at Cranleigh; and when Dr. James Maurice began work at Marlborough the Savernake Cottage Hospital was soon established. So successful did it prove that a new hospital was opened six years later, and has ever since been proof of the excellent and progressive work these small hospitals can do when they are blessed with the services of a highly trained and competent medical staff. We went from the funeral to the Savernake Hospital to satisfy ourselves as to the condition of that hospital as it exists to-day. It is enough to record that out of twenty patients in the beds at least sixteen were surgical cases which had required at the hands of the staff in every instance a major operation. In fact, any surgeon who wished for clinical material for his class could have found nothing better in any hospital in the country. This we take to be a striking confirmation of the confidence placed in him as a surgeon by his patients, the profession, and the public. It further proves the enormous advantage of atmosphere and example, for two out of three of the present staff of the hospital are sons of the late James Maurice, the third being Mr. T. H. Haydon, M.B., B.C.

The late James Blake Maurice not only made the Cottage Hospital, with his son's assistance, so efficient as to enable all the major surgical operations to be performed within its walls, but he maintained the great position in the county which his father occupied at the time of his death. The family doctor of half the country-side, fond of his profession, of farming, and of local affairs, several times Mayor of Marlborough, a county magistrate, and the trusted friend of all his neighbours, he spent a full and most useful life. From his earliest days he took the greatest pride in his profession, and a new course of treatment, a valuable life saved, and an up-to-date operation were to him sources of joy and congratulation. It is recorded of him that he loved his farm, he loved his fellow man, and he never forgot an old friend.

Here, then, we have presented on succeeding days two great contrasts—the eminent surgeon and scientist on the one hand, and the great general practitioner and surgeon on the other. The memory of the first was commemorated in the most impressive manner in the Abbey of Westminster. The second found his resting-place in a simple country churchyard in the centre of the district where his life work had been accomplished. The work of both men was excellent. The example they set their fellows was great and far-reaching, and the admiration and confidence which each inspired were evidenced in an equally striking and touching manner. Preshute Church was crowded with men representing the local corporate bodies, the magistrates, county magnates and gentry, and all classes from the highest to the humblest dwellers in and around Marlborough. The churchyard was equally crowded with many hundreds of people—men, women, and children, all of whom recognised the value to them of the life which had departed, and the loss of a friend and protector which they individually felt. Contrast the great Abbey, with all its historical and wonderful associations, its perfection of ritual and music, and all the grandeur of its architecture and many beauties, with the simple village church in Wilts. Then realise that as at the Abbey so in the church there was a silence of reverence, affection, and homage for the departed which could be felt. The service was as simple as it was suitable to the occasion. The music was supplied by the congregation, who in the most impressive and solemn way sang the hymns with such earnestness, feeling, and harmonious unity that the voices warmed the heart and stimulated the affections, and made the whole countryside feel this spontaneous living testimony to the value and influence for good of a long life spent largely in the service of others.

We have endeavoured to bring out convincingly, by a description of the unison and perfection of harmony which everywhere unites all, from the highest to the lowest, on occasions such as these, that, when the strain and stress of life is ended, there remains after death in the case of really useful lives the influence of everything that has been great and good in the work of the departed. It will encourage and comfort every one of us who labour for the sick, whoever we may be, however high or however humble our position in the ranks of workers, ever to keep this fact before us when the stress is hardest. Such remembrance should prove an inspiration and encouragement when we are most conscious of the joy of living and the opportunity which life affords to regard every day as doomsday. Thus haply we may each go about our Master's business, ever forgetful of self and always pushing forward to the further equipment of our individuality for better and higher work and for the fuller discharge of every responsibility which it may be our privilege to have entrusted to us.

